# Infection with tungiasis through interhost movement of adult female sand fleas, *Tunga penetrans*

**DOI:** 10.1093/trstmh/trab117

**Published:** 2022-02-01

**Authors:** Lynne Elson, Marlene Thielecke, Ulrike Fillinger, Hermann Feldmeier

**Affiliations:** a Centre for Tropical Medicine and Global Health, Nuffield Department of Medicine, University of Oxford, UK and KEMRI-Wellcome Trust, Kilifi 80108, Kenya; b Institute of Tropical Medicine and International Health, Charité University Medicine, Berlin 10117, Germany; c Human Health Theme, International Centre of Insect Physiology and Ecology, Nairobi 00100, Kenya; d Institute of Microbiology and Infectious Diseases Immunology, Charité University Medicine, Berlin, Germany

**Keywords:** tungiasis, *Tunga penetrans*

Tungiasis is a highly neglected tropical skin disease caused by the parasitic adult female sand flea, *Tunga penetrans.* More than 80% of tungiasis patients are found in the age group <15 y, in the elderly population and in people with disabilities. Tungiasis is a public health threat in the most marginalized, resourcepoor communities of sub-Saharan Africa, South America and the Caribbean.^1^ Patients struggle to walk, and their sleep is disturbed due to itching and pain. Children avoid going to school and hesitate to play with their friends because walking is painful. Their quality of life is significantly impaired,^2^ especially when constant re-infection leads to chronic clinical manifestations including desquamation, hyperkeratosis, fissures, ulcers, lymphoedema and loss of nails and deformation of toes.^1^ Bacterial superinfection is common, exacerbating the inflammation and pain.^1^

The cause for all this suffering is the unique lifestyle of the adult female sand flea that burrows into the epidermis and once fully embedded after approximately 6–8 h, starts to grow over a period of 7 d until it reaches the size of a pea. This process is known as neosomy and serves in the development of hundreds of eggs in the flea’s expanded abdomen.^3^ It is this growth of the parasite that causes the intense inflammation, pain and itching.^1^

Little entomological research has been implemented to date to fully understand the factors supporting the life cycle of *T. penetrans* as well as those supporting the distribution of this parasite. The off-host cycle is initiated when eggs are expelled from the gravid female and drop on the ground where, under optimal conditions, larvae hatch after 3–4 d. The larvae develop in the upper surface layer of loose soil, sand and dust similar to most other species in the flea family (Siphonaptera).^3^ The larval development takes at least 7 d, after which the larvae pupate and it takes another 7–9 d before adult fleas emerge to infect the next host.^3^

Several studies in different countries, have identified demographic, behavioural and environmental factors intricately associated with poverty as a risk for heavy infection, including sleeping in rooms or attending classrooms with soil floors and not washing feet frequently with soap.^4^ *T. penetrans* mostly parasitises the feet, but can also be found on the fingers, elbows and backs of patients who are severely restricted in their mobility.^5^ This is a clear indicator of the close association between the floor where the off-host stages develop and the host. Most flea species breed close to the resting and sleeping places of their hosts, in sand, soil, dust, dirt and cracks in floors, and *T. penetrans* is no exception. This association assures constant re-infection within the living quarters of the host.

Here we report an observation from the field that might aid in understanding how sand fleas, and therefore tungiasis, expands their distribution in space. All authors experienced once or repeatedly becoming infected by a female sand flea while conducting close observations of embedded fleas, sometimes placing one foot of the patient on their knees. During these times investigators wore closed shoes and were situated on cleanly swept, unbroken concrete floors where the flea, which penetrated the foot of the investigator, was unlikely to have developed. We frequently experienced during these close observations, live adult fleas running across the skin of the patient’s feet. These included both males, who were observed to mate with the embedded females, and females looking for a suitable site to penetrate (see Supplementary video S1). During a medical exam of a severely infected individual, one of the authors collected more than a dozen free-living male and female sand fleas from the skin.

From our observations, we suggest that people not only become infected from areas where the off-host stages develop, but also through interhost movement of adult female sand fleas when in close contact with severely infected individuals. Conditions of crowding might support this movement. Crowded classrooms, for example, might contribute to the spatial distribution of this disease within a village. Children sitting in close proximity to a heavily infected child might become infected and spread the disease to new households. In rural African schools it is not unusual for up to 4 pupils to share a desk and for up to 100 children to be confined for several hours in a classroom.

Based on the observations reported here, we propose further research into the behavioural ecology of *T. penetrans,* with special emphasis on host finding and selection cues and the time the free-living female parasite spends on the host skin before burrowing into the epidermis. Furthermore, establishing the frequency of interhost movements and the role of crowded conditions and how these affect disease distribution will provide important epidemiological insights.We recommend that public health departments and schools in disease-endemic areas take responsibility for treating all infected pupils at regular intervals and thus protect all pupils and potentially prevent the spread of the disease to a wider community. Any such action must be undertaken sensitively to avoid worsening the stigma associated with infection.

## Supplementary Material

S1. Video of an adult female *Tunga penetrans* attempting to penetrate the skin. © Marlene Thielecke https://doi.org/10.7910/DVN/E6IFU1

## Data Availability

There is no data set for the observations described in this manuscript.
